# Topological phenomena in honeycomb Floquet metamaterials

**DOI:** 10.1007/s00208-023-02583-0

**Published:** 2023-03-04

**Authors:** Habib Ammari, Thea Kosche

**Affiliations:** Department of Mathematics, ETH Zürich, Rämistrasse 101, 8092 Zurich, Switzerland

**Keywords:** 35J05, 35C20, 35P20, 74J20

## Abstract

Being driven by the goal of finding edge modes and of explaining the occurrence of edge modes in the case of time-modulated metamaterials in the high-contrast and subwavelength regime, we analyse the topological properties of Floquet normal forms of periodically parameterized time-periodic linear ordinary differential equations $\left\{ \frac{d}{dt}X = A_\alpha (t)X\right\} _{\alpha \in {\mathbb {T}}^d}$. In fact, our main goal being the question whether an analogous principle as the bulk-boundary correspondence of solid-state physics is possible in the case of Floquet metamaterials, i.e., subwavelength high-contrast time-modulated metamaterials. This paper is a first step in that direction. Since the bulk-boundary correspondence states that topological properties of the bulk materials characterize the occurrence of edge modes, we dedicate this paper to the topological analysis of subwavelength solutions in Floquet metamaterials. This work should thus be considered as a basis for further investigation on whether topological properties of the bulk materials are linked to the occurrence of edge modes. The subwavelength solutions being described by a periodically parameterized time-periodic linear ordinary differential equation $\left\{ \frac{d}{dt}X = A_\alpha (t)X\right\} _{\alpha \in {\mathbb {T}}^d}$, we put ourselves in the general setting of periodically parameterized time-periodic linear ordinary differential equations and introduce a way to (topologically) classify a Floquet normal form *F*,  *P* of the associated fundamental solution $\left\{ X_\alpha (t) = P(\alpha ,t)\exp (tF_\alpha )\right\} _{\alpha \in {\mathbb {T}}^d}$. This is achieved by analysing the topological properties of the eigenvalues and eigenvectors of the monodromy matrix $X_\alpha (T)$ and the Lyapunov transformation $P(\alpha ,t)$. The corresponding topological invariants can then be applied to the setting of Floquet metamaterials. In this paper these general results are considered in the case of a hexagonal structure. We provide two interesting examples of topologically non-trivial time-modulated hexagonal structures.

## Motivation and introduction

The study of topological properties in periodic physical systems is one of the most active fields in solid-state physics. The topological invariants are the building elements of topological band theory since they imply the presence of non-trivial bulk topologies, giving rise to topologically protected edge modes [7–9, 13, 24, 35, 37, 45]. Topological properties of electronic structures have been mathematically studied in the setting of the Schrödinger operator [10, 12, 15–18, 27]. Topological invariants have been defined to capture the crystal’s properties. Then, if part of a crystalline structure is replaced with an arrangement that is associated with a different value of this invariant, not only will certain modes be localized to the interface but this behaviour will be stable with respect to imperfections. These eigenmodes are known as *edge modes* and we say that they are *topologically protected* to refer to their robustness. This principle is known as the *bulk-boundary correspondence* in quantum settings [10, 11, 13, 20–22, 33]. Here, the term *bulk* is used to refer to parts of a crystal that are away from an edge.

Taking inspiration from quantum mechanics, subwavelength topological photonic and phononic crystals, based on locally resonant crystalline structures with large material contrasts, have been studied both numerically, experimentally, and mathematically in [3, 4, 14, 28–30, 32, 36, 38, 42–44, 46]. Subwavelength crystals (called also metamaterial structures) allow for the manipulation and localization of waves on very small spatial scales (much smaller than the wavelength) and are therefore very useful in physical applications, especially situations where the operating wavelengths are very large. Recently, this field has experienced tremendous advances by exploring the novel and promising area of time-modulations. Research on *Floquet metamaterials* (or time-modulated metamaterials) aims to explore new phenomena arising from the temporal modulation of the material parameters of the structure. It has enabled to open new paradigms for the manipulation of wave-matter interactions in both spatial and temporal domains [5, 19, 23, 25, 26, 31, 34, 39, 39–41]. To the best of our knowledge, the mathematical analysis of wave propagation properties of Floquet metamaterials has been just started. In [5], a discrete characterization of the band structure in Floquet metamaterials is introduced. This characterization provides both theoretical insight and efficient numerical methods to compute the dispersion relationship of time-dependent structures. A study of exceptional points in the case of Floquet metamaterials is conducted in [6]. Furthermore, in [1] the possibility of achieving non-reciprocal wave propagation in Floquet metamaterials is proven.

We are interested in edge modes, particularly edge modes which appear in honeycomb (e.g. graphene-like) structures and which result from topological non-trivialities due to time-modulation of the material parameters. In [5], it is shown that the subwavelength solutions to periodically time-modulated metamaterials are described by a second order linear ordinary differential equation (lODE). Using this result in the case of infinite periodic crystals, it follows that the subwavelength solutions are described by a periodically parameterized lODE $\left\{ \frac{d}{dt}X = A_\alpha (t)X\right\} _{\alpha \in {\mathbb {T}}^d}$ with time-periodic coefficient matrix $A_\alpha (t)$ and parameter space ${\mathbb {T}}^d \cong {\mathbb {S}}^1 \times \cdots \times {\mathbb {S}}^1$ (*d*-times) denoting the *d*-dimensional torus. This motivated the study of general periodically parameterized periodic lODEs of the form

1 $$\begin{aligned} \left\{ \frac{d}{dt}X = A_\alpha (t)X\right\} _{\alpha \in {\mathbb {T}}^d}, \end{aligned}$$

where $A_\alpha$ is periodic with respect to time *t*, say with a period *T*.

In this paper, we will first derive topological invariants which distinguish different topological properties in the case of a general periodically parameterized periodic lODE, see Sect. 2. To this end, the existence of continuously parameterized Floquet normal form is proven in Sect. 2.2. A continuously parameterized Floquet normal form associated to a given periodically parameterized periodic lODE then serves as a basis for the definition of the introduced topological invariants. In fact, we will distinguish between two types of topological invariants, to which we will refer to as Type I and Type II. The reason being that the Type I topological invariants are also meaningful in the setting of continuously parameterized *constant* lODEs, whereas non-trivial Type II topological invariants arise solely in the setting of time-dependent continuously parameterized periodic lODEs. The topological invariants are derived in Sect. 2.3.

In Sect. 3, these results are then applied to graphene-like Floquet metamaterials. First the precise setting from [5] is recalled in Sect. 3.1. Due to the symmetry of honeycomb structures the parameter space will be reduced from a two-dimensional torus ${\mathbb {T}}^2$ to a one-dimensional torus, that is, to the circle ${\mathbb {S}}^1$. The low dimension of the parameter space restricts the variety of meaningful topological invariants. The respective results are presented in Sect. 3.3. Finally the results will be applied to honeycomb Floquet metamaterials and two interesting examples will be presented in Sect. 3.4.

## Topological phenomena of periodically parameterized linear ODEs

The goal of this section is to investigate some topological phenomena arising in the case of periodically parameterized (with respect to $\alpha$) periodic lODEs $\{\frac{d}{dt}X = A(\alpha ,t)X\}_{\alpha \in {\mathbb {T}}^d}$, where $A(\alpha ,t)$ is periodic with respect to *t* with period *T* which is independent of $\alpha \in {\mathbb {T}}$. This will be achieved through an analysis of the Floquet normal form associated to the corresponding parameterized fundamental solution $\{X_\alpha (t)\}_{\alpha \in {\mathbb {T}}^d}$ and where ${\mathbb {T}}^d$ is regarded as ${\mathbb {R}}^d/L$, where $L \subset {\mathbb {R}}^d$ is a full-dimensional lattice. To this end, we will first prove the existence of a continuously differentiable Floquet normal form associated to the parameterized lODE $\{\frac{d}{dt}X = A(\pi (\gamma ),t)X\}_{\gamma \in {\mathbb {R}}^d}$, where $\pi$ is the canonical projection $\pi :{\mathbb {R}}^d \rightarrow {\mathbb {R}}^d/L = {\mathbb {T}}^d$. Under some assumptions on *A* and since ${\mathbb {R}}^d$ is simply connected, it will be possible to construct a continuously differentiable Floquet normal form $X_{\pi (\gamma )} = P(\gamma ,t)\exp (tF_\gamma )$ associated to the parameterized fundamental solution $X_{\pi (\gamma )}(t)$, $\gamma \in {\mathbb {R}}^d$. Analysis of the ${\mathbb {R}}^d$-parameterized Floquet normal form will lead to the definition of two topological invariants which will be called *Type I* and *Type II topological invariants*.

### Setting

Let $A: {\mathbb {R}}^d\times {\mathbb {R}} \rightarrow {{\,\mathrm{{Mat}}\,}}_{N\times N}({\mathbb {C}}), (\gamma , t) \rightarrow A(\gamma ,t)$ be a continuously differentiable function, which is periodic with respect to $(\gamma ,t)\in {\mathbb {R}}^{d+1}$. That is, there is a maximal lattice $L \subset {\mathbb {R}}^{d}$ and a positive number $T>0$, such that translations by lattice vectors of $L \times m{\mathbb {Z}}$ leave *A* invariant. Denoting by ${\mathbb {T}}^d$ the *d*-dimensional torus given by ${\mathbb {R}}^d/L$ and by ${\mathbb {S}}^1$ the circle ${\mathbb {R}}/T{\mathbb {Z}}$, the function *A* can be factored in the following waywhere $\pi : {\mathbb {R}}^{d} \rightarrow {\mathbb {T}}^{d}$ is given by $\pi (x) = x\mod L$ and $\psi : {\mathbb {R}} \rightarrow {\mathbb {S}}^{1}$ is given by $\psi (t) = t \mod T$. In what follows, the map $A: {\mathbb {R}}^d\times {\mathbb {R}} \rightarrow {{\,\mathrm{{Mat}}\,}}_{N\times N}({\mathbb {C}})$ and the associated map ${\tilde{A}}: {\mathbb {T}}^{d} \times {\mathbb {S}}^1 \rightarrow {{\,\mathrm{{Mat}}\,}}_{N\times N}({\mathbb {C}})$ will both be denoted by *A*. It will be distinguished between *A* and ${\tilde{A}}$ by using different notation for the argument, that is, $A(\gamma ,t)$ will indeed denote $A(\gamma ,t)$ where *A* is given by $A: {\mathbb {R}}^d\times {\mathbb {R}} \rightarrow {{\,\mathrm{{Mat}}\,}}_{N\times N}({\mathbb {C}})$ with $\gamma \in {\mathbb {R}}^d$ and $t \in {\mathbb {R}}$, $A(\alpha ,{\overline{t}})$ will denote ${\tilde{A}}(\alpha , {\overline{t}})$ with $\alpha \in {\mathbb {T}}^d$, ${\overline{t}} \in {\mathbb {S}}^1$ and $A(\alpha ,t)$ will denote ${\tilde{A}}(\alpha , {\overline{t}})$ where ${\overline{t}} = t \mod T$ with $\alpha \in {\mathbb {T}}^d$ and $t \in {\mathbb {R}}$.

In the following the topological invariants associated to the parameterized fundamental solution of the parameterized lODE

2 $$\begin{aligned} \left\{ \frac{d}{dt}X = A(\alpha ,t)X\right\} _{\alpha \in {\mathbb {T}}^d} \end{aligned}$$

will be defined and investigated. To this end, the family of fundamental solutions $\{X_\alpha \}_{\alpha \in {\mathbb {T}}^d}$ to the parameterized lODE (2) will be understood as a function

$$\begin{aligned}&X : {\mathbb {T}}^d\times {\mathbb {R}} \longrightarrow {{\,\textrm{GL}\,}}_{N}({\mathbb {C}})\\&\qquad (\alpha ,t) \longmapsto X_\alpha (t). \end{aligned}$$

In the following it will also be assumed that $X_{\alpha }(T)$ is diagonalizable for all $\alpha \in {\mathbb {T}}^{d}$ and such that for each $\alpha \in {\mathbb {T}}^d$ there exists a neighborhood $W \subset {\mathbb {T}}^d$ of $\alpha$ and continuously differentiable functions $\lambda _1,\ldots ,\lambda _N: W \rightarrow {\mathbb {C}}$ and $\eta _1,\ldots ,\eta _N: W \rightarrow {{\mathbb {C}}}{{\mathbb {P}}}^{N-1}$, such that for all $\alpha ' \in W$ the *N*-tuple $(\lambda _1(\alpha '),\ldots ,\lambda _N(\alpha '))$ gives the eigenvalues of $X_{\alpha '}(T)$ and $(\eta _1(\alpha '),\ldots ,\eta _N(\alpha '))$ are the respective eigenspaces. We will call this property *local* $C^1$*-diagonalizability*.^1^

### Continuously differentiably parameterized Floquet normal form

In the following subsection, the existence of a continuously differentiably parameterized Floquet normal form associated to $C^1$-diagonalizable fundamental solutions of a parameterized periodic lODE

3 $$\begin{aligned} \left\{ \frac{d}{dt}X = A(\gamma ,t)X\right\} _{\gamma \in {\mathbb {R}}^d} \end{aligned}$$

will be proven. This existence will then be used in the subsequent sections to analyse topological phenomena arising in the case of periodically parameterized periodic lODEs.

Given a periodic linear ordinary differential equation

4 $$\begin{aligned} \frac{d}{dt}X = A(t)X \end{aligned}$$

with $A: {\mathbb {R}} \rightarrow {{\,\mathrm{{Mat}}\,}}_{N \times N}({\mathbb {R}})$ being a *T*-periodic function, denote by *X* its associated fundamental solution, that is, the solution to the initial value problem

$$\begin{aligned} \frac{d}{dt}X&= A(t)X, \\ X(0)&= {{\,\textrm{Id}\,}}_N. \end{aligned}$$

Then Floquet theory states that *X* can be decomposed into a *Floquet normal form* which is a decomposition of the form

$$\begin{aligned} X(t) = P(t)\exp (tF), \end{aligned}$$

where $P: {\mathbb {R}} \rightarrow {{\,\textrm{GL}\,}}_N({\mathbb {C}})$ is *T*-periodic and $F \in {{\,\mathrm{{Mat}}\,}}_{N \times N}({\mathbb {C}})$ is a constant matrix. Note that ‘the’ Floquet normal form associated to the fundamental solution of *X* is never well-defined due to the non-injectivity of the exponential map. In fact, the eigenvalues of *F* are uniquely defined up to adding an integer multiple of $2\pi i/T$.

In the following we will refer to any choice of *F* as a *Floquet exponent matrix* associated to *X* or the lODE (4) and to the corresponding choice of *P* as the associated *Lyapunov transform*.

This choice of naming is due to the fact that a Lyapunov transform *P* associated to the lODE (4) can serve to transform the time-dependent lODE (4) into a lODE with constant coefficients. In fact, making the substitution $Y(t) = P(t)^{-1}X(t)$ will transform the time-dependent lODE (4) into the following lODE:

$$\begin{aligned} \frac{d}{dt}Y = F Y, \end{aligned}$$

where *F* is the Floquet exponent matrix corresponding to *P*.

One possibility to obtain a choice of Floquet normal form (even all choices of Floquet normal forms) associated to the periodic lODE (4) is to diagonalize the *monodromy matrix* *X*(*T*) of the lODE (4):

$$\begin{aligned} X(T) = \eta \Lambda \eta ^{-1} = \begin{pmatrix} | &{} &{}| \\ \eta _1 &{}\ldots &{}\eta _N \\ | &{} &{}| \\ \end{pmatrix}\begin{pmatrix} \lambda _1 &{} &{} \\ &{}\ddots &{} \\ &{} &{}\lambda _N \\ \end{pmatrix}\begin{pmatrix} | &{} &{}| \\ \eta _1 &{}\ldots &{}\eta _N \\ | &{} &{}| \\ \end{pmatrix}^{-1}. \end{aligned}$$

The eigenvalues $(\lambda _1,\ldots ,\lambda _N)$ are than the *characteristic multipliers* of the system (4) and all possible choices of *Floquet exponents* are in bijection with the solutions $(\mu _1,\ldots ,\mu _N)$ of the equation

$$\begin{aligned} \begin{pmatrix} \lambda _1 &{} &{} \\ &{}\ddots &{} \\ &{} &{}\lambda _N \\ \end{pmatrix} = \begin{pmatrix} \exp (T\mu _1) &{} &{} \\ &{}\ddots &{} \\ &{} &{}\exp (T\mu _N) \\ \end{pmatrix}. \end{aligned}$$

Given a choice of Floquet exponents $(\mu _1,\ldots ,\mu _N)$ the corresponding Floquet exponent matrix can be obtained via

$$\begin{aligned} F = \eta \mu \eta ^{-1} = \begin{pmatrix} | &{} &{}| \\ \eta _1 &{}\ldots &{}\eta _N \\ | &{} &{}| \\ \end{pmatrix}\begin{pmatrix} \mu _1 &{} &{} \\ &{}\ddots &{} \\ &{} &{}\mu _N \\ \end{pmatrix}\begin{pmatrix} | &{} &{}| \\ \eta _1 &{}\ldots &{}\eta _N \\ | &{} &{}| \\ \end{pmatrix}^{-1} \end{aligned}$$

Any choice of *F* and in particular the above choice of *F* uniquely determines the Lyapunov transform *P* as

$$\begin{aligned} P(t) = X(t)\exp (-tF), \end{aligned}$$

giving one possible choice of Floquet normal form associated to the periodic lODE (4).

In fact, one obtains all possible Floquet normal forms via the above procedure. Indeed, it is easy to see that the family of possible Floquet normal forms is in bijection with

$$\begin{aligned} \left\{ \begin{pmatrix} \mu _1 + n_1\frac{2\pi i}{T} &{} &{} \\ &{}\ddots &{} \\ &{} &{}\mu _N + n_N\frac{2\pi i}{T} \\ \end{pmatrix} \quad : \qquad n_1,\ldots , n_N \in {\mathbb {Z}} \right\} . \end{aligned}$$

We are interested in continuously differentiably parameterized Floquet normal forms

$$\begin{aligned} X(\gamma ,t) = P(\gamma ,t)\exp (tF_\gamma ) \end{aligned}$$

associated to parameterized periodic lODEs as in Eq. (3). To this end, we will use the above procedure which insures that any continuously differentiable parameterization of a choice of Floquet exponents $(\mu _1(\gamma ),\ldots ,\mu _N(\gamma ))$ and any continuously differentiable parameterization of a choice of eigenspaces $(\eta _1(\gamma ),\ldots ,\eta _N(\gamma ))$ provides a continuously differentiable parameterization of a choice of Floquet normal form of the system (3).

Given a parameterized time-periodic lODE of the form (as in Eq. (3))

$$\begin{aligned} \left\{ \frac{d}{dt}X = A(\gamma ,t)\right\} _{\gamma \in {\mathbb {R}}^d}, \end{aligned}$$

where $A(\gamma ,\cdot ): {\mathbb {R}} \rightarrow {{\,\mathrm{{Mat}}\,}}_{N \times N}({\mathbb {C}}^d)$ is *T*-periodic for all $\gamma \in {\mathbb {R}}^d$, we will thus need to construct continuously differentiable parameterizations of the Floquet exponents and corresponding eigenspaces of the Monodromy matrix. To this end, the following definition will become useful and is in fact, necessary for the construction of the desired continuously differentiable parametrizations.

#### Definition 1

(*local* $C^1$-*diagonalizability*) Let ${\mathcal {M}}$ be a differentiable manifold and let $M:{\mathcal {M}} \rightarrow {{\,\mathrm{{Mat}}\,}}_{N \times N}({\mathbb {C}})$ be a matrix-valued function. Then *M* is *locally* $C^1$-*diagonalizable* if the following holds. For all $\gamma \in {\mathcal {M}}$ there exists a neighborhood $W \subset {\mathcal {M}}$ of $\gamma$ and continuously differentiable functions $\lambda _1,\ldots ,\lambda _N: W \rightarrow {\mathbb {C}}$ and $\eta _1,\ldots ,\eta _N: W \rightarrow {{\mathbb {C}}}{{\mathbb {P}}}^{N-1}$, such that for all $\gamma ' \in W$ the *N*-tuple $(\lambda _1(\gamma '),\ldots ,\lambda _N(\gamma '))$ gives the eigenvalues of $X_{\gamma '}(T)$ and $(\eta _1(\gamma '),\ldots ,\eta _N(\gamma '))$ the respective eigenspaces of $M(\gamma ')$, such that the ${\mathbb {C}}$-span of the vector spaces $\eta _1(\gamma '),\ldots ,\eta _N(\gamma ')$ is equal to ${{\,\mathrm{{span}}\,}}_{\mathbb {C}}(\eta _1(\gamma '),\ldots ,\eta _N(\gamma ')) = {\mathbb {C}}^N$.

The following lemma will be at the heart of the existence of a continuously differentiably parametrized Floquet normal form. A continuously differentiably parametrized Floquet normal form is essential for the definition and well-posedness of the topological invariants defined in this paper. In the setting of high-contrast hexagonal structures (Sect. 3) this assumption corresponds to the absence of exceptional points in the system.

#### Lemma 1

Let $M: {\mathbb {R}}^d \rightarrow {{\,\mathrm{{Mat}}\,}}_{N\times N}({\mathbb {C}})$ be continuously differentiable and locally $C^1$-diagonalizable function. Denote by

$$\begin{aligned} \Lambda : \; {\mathbb {R}}^d \longrightarrow {\mathbb {C}}^N \end{aligned}$$

a map that associates to each $\gamma \in {\mathbb {R}}^d$ the eigenvalues $\Lambda (\gamma )=(\lambda _1(\gamma ), \ldots , \lambda _N(\gamma ))$ of $M(\gamma )$. Furthermore, denote by

$$\begin{aligned} \eta : \; {\mathbb {R}}^d \longrightarrow ({{\mathbb {C}}}{{\mathbb {P}}}^{N-1})^N \end{aligned}$$

the map that associates to each $\gamma \in {\mathbb {R}}^d$ the eigenspaces $\eta (\gamma )=(\eta _1(\gamma ), \ldots , \eta _N(\gamma ))$ of $M(\gamma )$, such that $\eta _n(\gamma )$ is the eigenspace corresponding to the eigenvalue $\Lambda _n(\gamma )$ and ${{\mathbb {C}}}{{\mathbb {P}}}^{N-1}$ denotes $(N-1)$-dimensional complex projective space.

Then the map $\Lambda \times \eta$ can be chosen to be continuously differentiable.

#### Proof

This is a direct consequence of the local $C^1$-diagonalizability of *M* and the simply connectedness of ${\mathbb {R}}^d$. $\square$

#### Theorem 1

$(C^1$-parameterized Floquet normal form) Let $A: {\mathbb {R}}^d \times {\mathbb {R}} \rightarrow {{\,\mathrm{{Mat}}\,}}_{N\times N}({\mathbb {C}})$ be continuously differentiable function such that

$$\begin{aligned} A(\gamma ,\cdot ) \text { is } T\text {-periodic for all } \gamma \in {\mathbb {R}}^d. \end{aligned}$$

Let $X_\gamma (t)$ be the fundamental solution associated to the parameterized lODE

5 $$\begin{aligned} \left\{ \frac{d}{dt}X = A(\gamma ,t)X\right\} _{\gamma \in {\mathbb {R}}^d}, \end{aligned}$$

and assume that the monodromy matrix

$$\begin{aligned} X(\cdot ,T): \quad&{\mathbb {R}}^d \longrightarrow \qquad {{\,\textrm{GL}\,}}_N({\mathbb {C}}),\\ \quad&\gamma \longmapsto \qquad X(\gamma ,T) \end{aligned}$$

is locally $C^1$-diagonalizable for all $\gamma \in {\mathbb {R}}^d$.

Then there exists a continuously differentiably parameterized Floquet normal form associated to the lODE (8). In fact, the following holds:

1. There exists a continuously differentiable parameterization of the characteristic multipliers
  $$\begin{aligned} (\lambda _1(\gamma ),\ldots ,\lambda _N(\gamma )) \end{aligned}$$
  and corresponding eigenspaces $(\eta _1(\gamma ),\ldots ,\eta _N(\gamma ))$ associated to the system (8).
2. Fixing such a continuously differentiable parametrization, there exists a choice of Floquet exponents $(\mu _1(\gamma ),\ldots ,\mu _N(\gamma ))$ which will satisfy $\exp (\mu _n T) = \lambda _n$ for $n \in \{1,\ldots ,N\}$ and which continuously differentiably depends on $\gamma \in {\mathbb {R}}^d$.
3. Fixing such a continuously differentiable parametrization, a continuously differentiably parameterized choice of Floquet normal form of the system (8) is given by
  $$\begin{aligned} F(\gamma )~ ~&= \begin{pmatrix} | &{} &{}| \\ \eta _1 &{}\ldots &{}\eta _N \\ | &{} &{}| \\ \end{pmatrix}\begin{pmatrix} \mu _1 &{} &{} \\ &{}\ddots &{} \\ &{} &{}\mu _N \\ \end{pmatrix}\begin{pmatrix} | &{} &{}| \\ \eta _1 &{}\ldots &{}\eta _N \\ | &{} &{}| \\ \end{pmatrix}^{-1}, \\ P(\gamma ,t)&= X_\gamma (t)\exp (-tF). \end{aligned}$$

#### Proof

The statement of the theorem mainly follows from the construction of a Floquet normal form in the beginning of this subsection together with Lemma 1 applied to the function $M:=X(\cdot ,T)$.

The first part is precisely the statement of Lemma 1, observing that the eigenvalues of $X(\cdot ,T)$ are precisely the characteristic multipliers and $\eta$ the corresponding eigenspaces.

In part two, the only thing that remains to be justified is the existence of a continuously differentiable choice of Floquet exponents associated to the given choice of characteristic multipliers $(\lambda _1(\gamma ),\ldots ,\lambda _N(\gamma ))$. Its existence is due to the fact that the exponential map gives a universal cover of the space ${\mathbb {C}}^*$ and due to the simply connectedness of ${\mathbb {R}}^d$, it is possible to lift the maps $\lambda _n: {\mathbb {R}}^d \rightarrow {\mathbb {C}}^*$ to maps $\mu _n: {\mathbb {R}}^d \rightarrow {\mathbb {C}}$ via the exponential map $\exp (T(\cdot ))$. In other words, for any $n \in \{1,\ldots ,N\}$ there exists a continuously differentiable map $\mu _n$ which makes the following diagram commute:Part three follows when the procedure, which was explained in the first half of this subsection, is applied to the continuously differentiable parameterization of a choice of Floquet exponents of part two and to the continuously differentiable parameterization of a choice of eigenspaces of part one. $\square$

### Topological phenomena of parameterized Floquet normal forms

In this subsection we will come back to the setting where the considered parameterized periodic lODE

6 $$\begin{aligned} \left\{ \frac{d}{dt}x = A(\alpha ,t)x \right\} _{\alpha \in {\mathbb {T}}^d} \end{aligned}$$

is periodically parameterized by a torus ${\mathbb {T}}^d$, which is understood as ${\mathbb {T}}^d = {\mathbb {R}}^d/L$ for some fixed lattice $L \subset {\mathbb {R}}^d$.

The parameterized system (6) can be rewritten as

$$\begin{aligned} \left\{ \frac{d}{dt}x = A(\gamma ,t)x \right\} _{\gamma \in {\mathbb {R}}^d} \end{aligned}$$

together with the identification $A(\gamma ,t) = A(\alpha ,t)$ for $\gamma \mod L = \alpha \in {\mathbb {T}}^d$.

Assume that the monodromy matrix associated to the system (6) is locally $C^1$-diagonalizable. Then it follows from Theorem 1 that there exist continuously differentiable parameterizations of the characteristic multipliers

$$\begin{aligned} (\lambda _n: {\mathbb {R}}^d \rightarrow {\mathbb {C}}^*)_{n = 1}^N \end{aligned}$$

with corresponding eigenspaces

$$\begin{aligned} (\eta _n: {\mathbb {R}}^d \rightarrow {{\mathbb {C}}}{{\mathbb {P}}}^{N - 1})_{n = 1}^N. \end{aligned}$$

Being eigenvalues and eigenvectors of the *L*-periodic map $X(T,\cdot )$, they fulfill the following property:

$$\begin{aligned} \gamma \in {\mathbb {R}}^d&\qquad \longmapsto \{\lambda _1(\gamma ), \ldots , \lambda _N(\gamma )\} \in \{A \subset {\mathbb {R}}^N\} \\ \gamma \in {\mathbb {R}}^d&\qquad \longmapsto \{\eta _1(\gamma ), \ldots , \eta _N(\gamma )\} \in \{A \subset {{\mathbb {C}}}{{\mathbb {P}}}^{N - 1}\} \end{aligned}$$

are also *L*-periodic. In other words, the set of eigenvalues $\{\lambda _1(\gamma ), \ldots , \lambda _N(\gamma )\}$ and the set of eigenspaces $\{\eta _1(\gamma ), \ldots , \eta _N(\gamma )\}$ are *L*-periodic. However, the maps $\lambda _n: {\mathbb {R}}^d \rightarrow {\mathbb {C}}^*$ do not need to be *L*-periodic. Since there are only finitely many eigenvalues and since the set of eigenvalues is *L*-periodic, it follows that for every $n \in \{1,\ldots ,N\}$ there exists a lattice $L_n \subset L$ such that $\lambda _n$ is $L_n$-periodic. Choosing this lattice to be a maximal sublattice $L_n \subset L$ such that $\lambda _n$ is $L_n$-periodic, makes the lattice $L_n$ unique and thus allows for the following well-defined topological invariant associated to the eigenvalue $\lambda _n: {\mathbb {R}}^d \rightarrow {\mathbb {C}}^*$.

#### Definition 2

(*Type I.a topological invariant*) Let

$$\begin{aligned} \left\{ \frac{d}{dt}x = A(\alpha ,t)x \right\} _{\alpha \in {\mathbb {T}}^d} \end{aligned}$$

be a periodically parameterized periodic lODE and let $A(\gamma ,\cdot )$ be *T*-periodic for all $\gamma \in {\mathbb {R}}^d$. If the monodromy matrix $X_\alpha (T)$ is locally $C^1$-diagonalizable and $(\lambda _1(\gamma ), \ldots , \lambda _N(\gamma ))$ is a continuously differentiable parameterization of the characteristic multipliers, then the *Type I.a topological invariant* associated to $\lambda _n$ is defined as the quotient

$$\begin{aligned} L/L_n, \end{aligned}$$

where $L \subset {\mathbb {R}}^d$ is the lattice associated to the parameter space ${\mathbb {T}}^d$ of the parameterized lODE and $L_n \subset L$ is the maximal sublattice of *L* such that $\lambda _n$ is $L_n$-periodic.

This topological effect indicates whether the different bands corresponding to the characteristic multipliers “brade”. We call *brading* the effect shown in Fig.  for the case where the parameter space is one-dimensional. Brading describes the effect when different bands $\lambda _n$ and $\lambda _{n'}$ are not *L*-periodic but only the set $\{\lambda _n(\gamma ),\lambda _{n'}(\gamma )\}$ is. In that case, we say that $\lambda _n$ and $\lambda _{n'}$ brade. This effect can involve multiple bands, see e.g. Sect. 3.4.

**Fig. 1:**
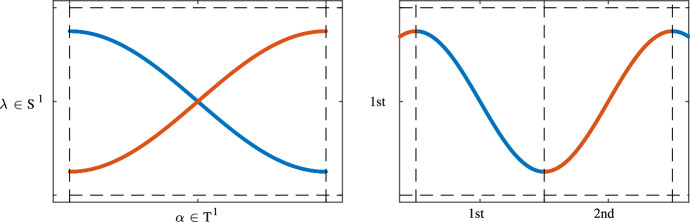
Non-trivial Type I.a topological invariant, also called brading. In the left figure a band structure parametrized with a one dimensional torus is depicted. One sees that the band structure is continuously parametrized with respect to the torus ${\mathbb {T}}^1 \cong {\mathbb {R}}/{\mathbb {Z}}$, however the individual bands $\lambda _1, \lambda _2$ *cannot* be continuously parameterized with respect to the torus ${\mathbb {T}}^1 \cong {\mathbb {R}}/{\mathbb {Z}}$. This is due to the fact that the bands *brade*. Looking at a lifting of the bands $\lambda _1, \lambda _2: {\mathbb {R}} \rightarrow {\mathbb {R}}$ (in the right figure) one sees that they glue together and actually form a 2-periodic band. Hence the maximal sublattice $L_1 = L_2$ such that $\lambda _1, \lambda _2$ are $L_1, L_2$-periodic is given by $L_1 = L_2 = 2{\mathbb {Z}}$. Thus the Type I.a topological invariant associated to $\lambda _1$ and $\lambda _2$ is given by ${\mathbb {Z}}/2{\mathbb {Z}}$

For a given band $\lambda _n$ with corresponding maximal sublattice $L_n \subset L$, it is possible to interpret it as well as its associated eigenspace $\eta _n$ as functions

$$\begin{aligned} \lambda _n :&\quad {\mathbb {R}}^d/L_n \longrightarrow \quad \quad {\mathbb {C}}^*, \\ \eta _n :&\quad {\mathbb {R}}^d/L_n \longrightarrow \quad \quad {{\mathbb {C}}}{{\mathbb {P}}}^{N-1}, \\ \end{aligned}$$

on the torus ${\mathbb {T}}^d_n:= {\mathbb {R}}^d/L_n$ and to consider the associated homotopy classes of those functions. This leads to the following two definitions.

#### Definition 3

(*Type I.b homotopy class*) Let

$$\begin{aligned} \left\{ \frac{d}{dt}x = A(\alpha ,t)x \right\} _{\alpha \in {\mathbb {T}}^d} \end{aligned}$$

be a periodically parameterized periodic lODE and let *T* be the period of $A(\gamma ,\cdot )$. If the monodromy matrix $X_\alpha (T)$ is locally $C^1$-diagonalizable and $(\lambda _1(\gamma ), \ldots , \lambda _N(\gamma ))$ is a continuously differentiable parameterization of the characteristic multipliers and $(\eta _1(\gamma ), \ldots , \eta _N(\gamma ))$ are the corresponding parameterized eigenspaces, then the *Type I.b homotopy class* associated to $\eta _n$ is defined as the homotopy class

$$\begin{aligned} \eta _n \in [{\mathbb {T}}^d_n, {{\mathbb {C}}}{{\mathbb {P}}}^{N-1}], \end{aligned}$$

where ${\mathbb {T}}^d_n$ is the torus associated to $\lambda _n$, that is, ${\mathbb {T}}^d_n$ is defined as ${\mathbb {R}}^d/L_n$ where $L_n \subset L$ is the maximal sublattice such that $\lambda _n$ is $L_n$-periodic.

#### Definition 4

(*Type II.a homotopy class*) Let

$$\begin{aligned} \left\{ \frac{d}{dt}x = A(\alpha ,t)x \right\} _{\alpha \in {\mathbb {T}}^d} \end{aligned}$$

be a periodically parameterized periodic lODE and let *T* be the period of $A(\gamma ,\cdot )$. If the monodromy matrix $X_\alpha (T)$ is locally $C^1$-diagonalizable and $(\lambda _1(\gamma ), \ldots , \lambda _N(\gamma ))$ is a continuously differentiable parameterization of the characteristic multipliers, then the *Type II.a homotopy class* associated to $\lambda _n$ is defined as the homotopy class

$$\begin{aligned} \lambda _n \in [{\mathbb {T}}^d_n, {\mathbb {C}}^*], \end{aligned}$$

where ${\mathbb {T}}^d_n$ is the torus associated to $\lambda _n$, that is, ${\mathbb {T}}^d_n$ is defined as ${\mathbb {R}}^d/L_n$ where $L_n \subset L$ is the maximal sublattice such that $\lambda _n$ is $L_n$-periodic.

The Type I and Type II terminology is due to the nature of the topological effects. Topological effects I.a and I.b can already occur in the case of a time-independent coefficient matrix $A(t,\gamma ) = A_{const}(\gamma )$, whereas Type II topological invariants are unique to the setting when the coefficient matrix $A(t,\gamma )$ is time-dependent.

The topological invariants which have been defined so far are all linked to the Floquet exponent matrix *F* or actually the monodromy matrix $X(T,\cdot )$ and are independent of the Lyapunov transformation $P(t,\gamma )$. However, the Type II.a homotopy class will indicate whether *P* can be defined periodically with respect to $\gamma$.

The reason why a non-trivial Type II.a invariant implies non-periodic Lyapunov transformation becomes clear when one considers a one-dimensional parameter set ${\mathbb {T}}^1$ of the parameterized periodic lODE $\{\frac{d}{dt}x = A(\alpha ,t)x\}_{\alpha \in {\mathbb {T}}^1}$. Assume $\lambda _n(\gamma )$ is a characteristic multiplier of the parameterized lODE and is $L_n$-periodic with $L_n \subset L$ and has non-trivial homotopy class $\lambda _n \in [{\mathbb {R}}/L_n, {\mathbb {C}}^*]$. This means that the image of the circle ${\mathbb {R}}/L_n$ winds around the origin in ${\mathbb {C}}^*$ and that is why we also say that the band associated to a characteristic multiplier *winds* or has *non-trivial winding* whenever that characteristic multiplier has non-trivial Type II.a.

**Fig. 2:**
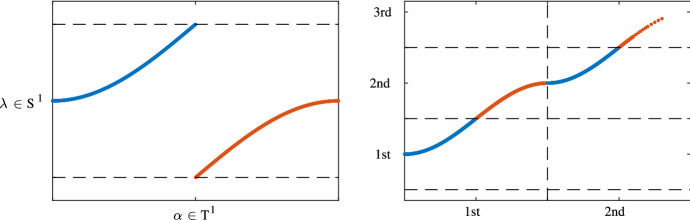
Non-trivial Type II.a topological invariant, also called winding. For the sake of simplicity, the band $\lambda : {\mathbb {T}}^1 \rightarrow {\mathbb {C}}^*$ is depicted in the left figure by its homotopic equivalent analogue $\lambda : {\mathbb {T}}^1 \rightarrow {\mathbb {S}}^1$ which takes its values in the unit circle ${\mathbb {S}}^1 \subset {\mathbb {C}}$ (that is, in the figure, the dashed horizontal lines are identified, such that the image of $\lambda$ lives indeed on a circle). One observes that $\lambda$ is homotopically *non-trivial* since it *winds* once around the circle. This becomes clear, when considering the right figure, where a lifting $\mu : {\mathbb {R}} \rightarrow {\mathbb {R}}$ of $\lambda$ is depicted. One observes that $\mu$ gains a phase with each fundamental domain that it traverses. That is $\mu (x + L) = \mu (x) + 2\pi$, where ${\mathbb {T}}^1 = {\mathbb {R}}/L{\mathbb {Z}}$ and ${\mathbb {S}}^1 = {\mathbb {R}}/2\pi {\mathbb {Z}}$

Winding has the following effect on the Floquet exponent matrix or more precisely on the characteristic exponents $\mu _n$. The characteristic exponents $\mu _n$ are obtained via lifting the characteristic multipliers $\lambda _n$ in the following wayA non-trivial winding of $\lambda _n$ will then lead to a non-periodic lift $\mu _n$. This phenomenon is depicted in Fig. , namely, $\mu _n$ gains a non-zero phase for every fundamental domain of $L_n$ that it traverses, leading to a linearly increasing characteristic exponent. This then implies that also the Floquet exponent matrix *F* will not be periodic but will have growing/decaying eigenvalues. Finally this property propagates to the Lyapunov transformation *P*. In the case of non-trivial winding also *P* will not be periodic with respect to $\gamma \in {\mathbb {R}}$.

Since, to our knowledge, the phenomenon of non-trivial winding in the case of subwavelength solutions for Floquet metamaterials hasn’t been observed yet, we will restrict this work to the case where the Type II.a homotopy class is trivial. A sufficient condition for this assumption to be valid would be that the coefficient matrix $A(\gamma ,t)$ commutes with its derivative $\frac{d}{dt}A(\gamma ,t)$ for all $(\gamma ,t) \in {\mathbb {R}}^{d+1}$. Indeed, if $A(\gamma ,t)$ and $\frac{d}{dt}A(\gamma ,t)$ commute for all $(\gamma ,t) \in {\mathbb {R}}^{d+1}$, then the fundamental solution to the system (6) is given by $X_\alpha (t) = \exp (\int _0^tA(\alpha ,s)ds)$, in which case the characteristic exponents $\mu _n$ are given by the eigenvalues of $\frac{1}{T}\int _0^TA(\alpha ,t)dt$, where *T* is the period of $A(\alpha ,\cdot )$ for all $\alpha \in {\mathbb {T}}^d$. As such the characteristic exponents $\mu _n$ are bounded, hence the characteristic multipliers $\lambda _n$ are homotopically trivial.

#### Lemma 2

(Periodic Lyapunov transform) Let $A: {\mathbb {R}}^d \times {\mathbb {R}} \rightarrow {{\,\mathrm{{Mat}}\,}}_{N\times N}({\mathbb {C}})$ be continuously differentiable function such that

$$\begin{aligned} A(\gamma ,\cdot ) \text { is } T\text {-periodic for all } \gamma \in {\mathbb {R}}^d \text { and such that } A(\cdot ,t) \text { is } L\text {-periodic for all } t \in {\mathbb {R}}, \end{aligned}$$

where $L \subset {\mathbb {R}}$ is a lattice. Let $X_\gamma (t)$ be the fundamental solution associated to the parameterized lODE

7 $$\begin{aligned} \left\{ \frac{d}{dt}X = A(\gamma ,t)\right\} _{\gamma \in {\mathbb {R}}^d}, \end{aligned}$$

and assume that the monodromy matrix

$$\begin{aligned} X(\cdot ,T): \quad&{\mathbb {R}}^d \longrightarrow \qquad {{\,\textrm{GL}\,}}_N({\mathbb {C}}),\\ \quad&\gamma \longmapsto \qquad X(\gamma ,t) \end{aligned}$$

is locally $C^1$-diagonalizable for all $\gamma \in {\mathbb {R}}^d$. Let $(\lambda _1(\gamma ),\ldots ,\lambda _N(\gamma ))$ denote a continuously differentiable parameterization of the characteristic multipliers with corresponding eigenspaces $(\eta _1(\gamma ),\ldots ,\eta _N(\gamma ))$ and maximal lattices $(L_1,\ldots ,L_N)$. Let $(\mu _1(\gamma ),\ldots ,\mu _N(\gamma ))$ be a continuously differentiable choice of characteristic exponents and assume that for all $n \in \{1,\ldots ,N\}$ the Type II.a of $\lambda _n$ is trivial. Then the associated Floquet exponent matrix $F(\gamma )$ and the corresponding Lyapunov transform $P(\gamma ,t)$ are ${\tilde{L}}$-periodic, where

$$\begin{aligned} {\tilde{L}} = \bigcap _{n=1}^N L_n. \end{aligned}$$

#### Proof

It needs to be proven that the $(\mu _1(\gamma ),\ldots ,\mu _N(\gamma ))$ defines a ${\tilde{L}}$-periodic map. Indeed, this being proven it follows from

$$\begin{aligned} F(\gamma )~ ~&= \begin{pmatrix} | &{} &{}| \\ \eta _1 &{}\ldots &{}\eta _N \\ | &{} &{}| \\ \end{pmatrix}\begin{pmatrix} \mu _1 &{} &{} \\ &{}\ddots &{} \\ &{} &{}\mu _N \\ \end{pmatrix}\begin{pmatrix} | &{} &{}| \\ \eta _1 &{}\ldots &{}\eta _N \\ | &{} &{}| \\ \end{pmatrix}^{-1} \text { and } \\ P(\gamma ,t)&= X_\gamma (t)\exp (-tF) \end{aligned}$$

that also *F* and *P* are ${\tilde{L}}$-periodic.

Let $n \in \{1,\ldots ,N\}$. Then $\mu _n$ is characterized by the equation $\exp (\mu _n) = \lambda _n$ and its value $\mu _n(1)$. Interpreting $\exp : {\mathbb {C}} \rightarrow {\mathbb {C}}^*$ as a universal cover, $\mu _n: {\mathbb {R}}^d \rightarrow {\mathbb {C}}$ is then given as a lifting of $\lambda _n: {\mathbb {R}}^d \rightarrow {\mathbb {C}}^*$. Since $\lambda _n$ is $L_n$-periodic and contractible, it follows that also $\mu _n$ is $L_n$-periodic. Thus proving that $(\mu _1(\gamma ),\ldots ,\mu _N(\gamma ))$ is ${\tilde{L}}$-periodic. $\square$

#### Definition 5

(*Type II.b homotopy class*) Let $A: {\mathbb {R}}^d \times {\mathbb {R}} \rightarrow {{\,\mathrm{{Mat}}\,}}_{N\times N}({\mathbb {C}})$ be continuously differentiable function such that

$$\begin{aligned} A(\gamma ,\cdot ) \text { is } T\text {-periodic for all } \gamma \in {\mathbb {R}}^d \text { and such that } A(\cdot ,t) \text { is } L\text {-periodic for all } t \in {\mathbb {R}}, \end{aligned}$$

where $L \subset {\mathbb {R}}$ is a lattice. Let $X_\gamma (t)$ be the fundamental solution associated to the parameterized lODE

8 $$\begin{aligned} \left\{ \frac{d}{dt}X = A(\gamma ,t)\right\} _{\gamma \in {\mathbb {R}}^d}, \end{aligned}$$

and assume that the monodromy matrix

$$\begin{aligned} X(\cdot ,T): \quad&{\mathbb {R}}^d \longrightarrow \qquad {{\,\textrm{GL}\,}}_N({\mathbb {C}}),\\ \quad&\gamma \longmapsto \qquad X(\gamma ,t) \end{aligned}$$

is locally $C^1$-diagonalizable for all $\gamma \in {\mathbb {R}}^d$. Let $(\lambda _1(\gamma ),\ldots ,\lambda _N(\gamma ))$ denote a continuously differentiable parameterization of the characteristic multipliers with corresponding eigenspaces $(\eta _1(\gamma ),\ldots ,\eta _N(\gamma ))$ and associated maximal lattices $(L_1,\ldots ,L_N)$. Let $(\mu _1(\gamma ),\ldots ,\mu _N(\gamma ))$ be a continuously differentiable choice of characteristic exponents and assume that for all $n \in \{1,\ldots ,N\}$ the Type II.a of $\lambda _n$ is trivial, then the *Type II.b homotopy class* associated to the corresponding Lyapunov transform *P* is defined as the homotopy class

$$\begin{aligned} P \in [{\mathbb {R}}^d/{\tilde{L}} \times {\mathbb {R}}/T{\mathbb {Z}}, {{\,\textrm{GL}\,}}_N({\mathbb {C}})], \end{aligned}$$

where ${\tilde{L}}:= \cap _{1=n}^N L_n$ is a lattice in ${\mathbb {R}}^d$.

#### Remark 1

The Type I.a and Type II.b topological invariants are trivial in the case of a system which is constant with respect to time. Indeed, assume that $A(\alpha ,t) = A(\alpha )$ is constant with respect to time *t*, then the fundamental solution to the system (6) is given by

$$\begin{aligned} X_\alpha (t) = \exp (A_\alpha t). \end{aligned}$$

Let $T > 0$ and interpret the system (6) as a system with period $T > 0$. In this case an associated Floquet exponent matrix $F_\alpha$ is given by $A_\alpha$ and thus the Lyapunov transform, given by $P(\gamma ,t) = X_\gamma (t)\exp (-F_\gamma t)$ is constant equal to the identity matrix $P(\gamma ,t) = Id_N$. Hence the Type II.b topological invariant is trivial. The triviality of the Type II.a topological invariant follows from the argument given before Lemma 2.

Using polar decomposition of matrices and the fact that the space of positive definite matrices is contractible, it is possible to rephrase the definition of the Type II.b homotopy class. Indeed, it suffices to examine the homotopy class of the unitary part $U(\gamma ,t)$ of $P(\gamma ,t)$ in

$$\begin{aligned} {[}{\mathbb {R}}^d/{\tilde{L}} \times {\mathbb {R}}/T{\mathbb {Z}}, {{\,\textrm{U}\,}}(N){]}, \end{aligned}$$

where ${{\,\textrm{U}\,}}(N)$ denotes the space of $N\times N$-dimensional unitary matrices. Denoting by ${{\,\textrm{SU}\,}}(N)$ the space of special unitary $N \times N$-dimensional matrices and using the diffeomorphism

$$\begin{aligned} {{\,\textrm{U}\,}}(N) \quad \quad \quad&\longrightarrow \qquad \qquad \qquad \quad {\mathbb {S}}^1 \times {{\,\textrm{SU}\,}}(N) \\ V \quad \quad \quad&\longmapsto \left( \det (V), \begin{pmatrix} | &{} | &{} &{}| \\ \frac{1}{\det (V)}V^{(1)} &{}V^{(2)} &{} \ldots &{}V^{(N)} \\ | &{} | &{} &{}| \end{pmatrix} \right) , \end{aligned}$$

the homotopy class of $P \in [{\mathbb {R}}^d/{\tilde{L}} \times {\mathbb {R}}/T{\mathbb {Z}}, {{\,\textrm{GL}\,}}_N({\mathbb {C}})]$ is uniquely determined by the associated homotopy classes in $[{\mathbb {R}}^d/{\tilde{L}} \times {\mathbb {R}}/T{\mathbb {Z}}, {\mathbb {S}}^1]$ and $[{\mathbb {R}}^d/{\tilde{L}} \times {\mathbb {R}}/T{\mathbb {Z}}, {{\,\textrm{SU}\,}}(N)]$. More precisely, the homotopy class of *P* is uniquely determined by the functions

$$\begin{aligned} \frac{\det (P(\gamma ,t))}{|\det (P(\gamma ,t))|} \in C^0({\mathbb {R}}^d/{\tilde{L}} \times {\mathbb {R}}/T{\mathbb {Z}}, {\mathbb {S}}^1) \end{aligned}$$

and

9 $$\begin{aligned}{} & {} S(\gamma ,t):=\nonumber \\{} & {} \begin{pmatrix} | &{} | &{} &{}| \\ \frac{|\det (P) |}{\det (P)}(P (P^*P)^{-\frac{1}{2}})^{(1)} &{}(P (P^*P)^{-\frac{1}{2}})^{(2)} &{} \ldots &{}(P (P^*P)^{-\frac{1}{2}})^{(N)} \\ | &{} | &{} &{}| \end{pmatrix}\nonumber \\ {}{} & {} \in C^0({\mathbb {R}}^d/{\tilde{L}} \times {\mathbb {R}}/T{\mathbb {Z}}, {{\,\textrm{SU}\,}}(N)), \end{aligned}$$

where $(P (P^*P)^{-\frac{1}{2}})^{(n)}$ denotes the *n*th column of $(P (P^*P)^{-\frac{1}{2}})^{(n)}$.

The following lemma will further simplify the homotopy class of $\det (P)/|\det (P)|$. Namely, it will be proven that the homotopy class of $\det (P)/|\det (P)|$ solely depends on $\det (P(\gamma ,\cdot ))/|\det (P(\gamma ,\cdot ))| \in C^0({\mathbb {R}}/T{\mathbb {Z}}, {\mathbb {S}}^1)$ for any choice of fixed $\gamma \in {\mathbb {R}}^d/{\tilde{L}}$.

#### Lemma 3

Let $A:{\mathbb {R}}^d \times {\mathbb {R}} \rightarrow {{\,\mathrm{{Mat}}\,}}_{N\times N}({\mathbb {C}})$ be a $L \times T{\mathbb {Z}}$-periodic, continuously differentiable function and assuming the setting of Lemma 2. Let $P: {\mathbb {R}}^d/{\tilde{L}} \times {\mathbb {R}}/T{\mathbb {Z}} \rightarrow {{\,\textrm{GL}\,}}_N({\mathbb {C}})$ be the Lyapunov transform obtained in Lemma 2 and define

$$\begin{aligned} \begin{array}{llll} \Delta :\quad \quad &{}{{\,\textrm{GL}\,}}_N({\mathbb {C}}) &{}\longrightarrow &{}\qquad {\mathbb {S}}^1\\ &{}M &{}\longmapsto &{}\quad \frac{\det (M)}{|\det (M)|},\\ \iota :\quad \quad &{}{\mathbb {S}}^1 &{}\longrightarrow &{}\qquad {\mathbb {T}}^{d+1}\\ &{}{\overline{t}} &{}\longmapsto &{}(0,\ldots ,0,{\overline{t}}). \end{array} \end{aligned}$$

Then the homotopy class of $\Delta \circ P = \frac{\det (P)}{|\det (P)|} \in C^0({\mathbb {R}}^d/{\tilde{L}} \times {\mathbb {R}}/T{\mathbb {Z}}, {\mathbb {S}}^1)$ is uniquely determined by $\Delta \circ P \circ \iota$ in $\pi ^1({\mathbb {S}}^1)$.

#### Proof

In fact, the homotopy class of a continuous map $f: {\mathbb {T}}^{d+1} \rightarrow {\mathbb {S}}^1$ is uniquely determined by the homotopy classes of $f\circ \iota _j: {\mathbb {S}}^{1} \rightarrow {\mathbb {S}}^1$, where $\iota _j: {\mathbb {S}}^1 \hookrightarrow {\mathbb {T}}^{d+1}\cong ({\mathbb {S}}^1)^{d+1}$ is the inclusion in the *j*th factor. Thus the homotopy class of $\Delta \circ P$ is also uniquely determined by the homotopy classes of the maps $\Delta \circ P \circ \iota _1, \ldots , \Delta \circ P \circ \iota _{d+1} \in C^0({\mathbb {S}}^1,{\mathbb {S}}^1)$. Since $P(\alpha ,0) = {{\,\textrm{Id}\,}}_N$ for all $\alpha \in {\mathbb {T}}^d$ it follows that the maps $\Delta \circ P \circ \iota _1, \ldots , \Delta \circ P \circ \iota _{d}$ are homotopically trivial and thus the homotopy class of $\Delta \circ P$ is uniquely determined by $\Delta \circ P \circ \iota _{d+1} = \Delta \circ P \circ \iota$. $\square$

#### Remark 2

This argument (i.e. Lemma 3 and the preceding discussion) also holds in the setting where *F* can be chosen to be real-valued. In that case, $P(\alpha ,t)$ takes values in ${{\,\textrm{GL}\,}}_N({\mathbb {R}})$ and one can replace ${{\,\textrm{U}\,}}(N)$ and ${{\,\textrm{SU}\,}}(N)$ by ${{\,\textrm{O}\,}}(N)$ and ${{\,\textrm{SO}\,}}(N)$ (the spaces of orthogonal and special orthogonal $N \times N$-dimensional matrices), respectively.

## Application to high-contrast hexagonal structures

In this section the derived topological invariants will be applied to the setting of a hexagonal structure Floquet metamaterial, like it is present in graphene. Highly symmetric structures as honeycomb or square lattice structures allow for a reduction of the two dimensional Brillouin zone to a one dimensional symmetry curve (see Fig. ). This then reduces the parameterization space from a two dimensional torus ${\mathbb {T}}^2$ to a one-dimensional circle ${\mathbb {S}}^1$, which in return limits the variety of homotopic effects, as will be displayed in Sect. 3.3.

In the following section we will present the setting for which we will apply the derived topological invariants. In the subsequent section we will briefly explain the computational procedure we use to compute the Floquet-Lyapunov decomposition, the Floquet exponents and modes. Thereafter, examples of a time-modulated honeycomb structures which have nontrivial Type I.a Topological Invariants will be presented.

### Setting

A metamaterial is a prototype material of the following form. It is composed of two submaterials: the *background* and the *resonator material*. While the background material fills almost the whole space ${\mathbb {R}}^d$, in this case we will restrict us to 2-dimensional space ${\mathbb {R}}^2$, the resonator material only occupies disconnected domains $D_1, \ldots , D_N \subset {\mathbb {R}}^2$, which are repeated periodically with respect to some ${\mathbb {R}}$-linearly independent lattice vectors $g_1, g_2 \in {\mathbb {R}}^2$, which generate the lattice $G = g_1{\mathbb {Z}} \oplus g_2{\mathbb {Z}}$, such that the domain of the resonator material is given by

$$\begin{aligned} {\mathcal {D}} =\bigcup _{g \in G}(g + D_1 \cup \cdots \cup D_N), \end{aligned}$$

see e.g. Fig.  for the case of a hexagonal structure material. The *reciprocal lattice* is then defined as

$$\begin{aligned} L := \left\{ l \in {\mathbb {R}}^2 | <l,g>\, \in 2\pi {\mathbb {Z}} \text { for all } g \in G \right\} , \end{aligned}$$

where $<l,g>$ denotes the standard scalar product in ${\mathbb {R}}^2$. Background and resonator material are characterized by their corresponding material parameters $\rho$ and $\kappa$ which correspond to the density and the bulk modulus in the setting of scalar (e.g. acoustic) waves. To be precise, the density $\rho$ and the bulk modulus $\kappa$ are defined as

10 $$\begin{aligned} \kappa (x,t)&= {\left\{ \begin{array}{ll} \kappa _0, &{} x \in {\mathbb {R}}^2 \setminus \overline{{\mathcal {D}}}, \\ \kappa _n(t), &{} x\in g + D_n,\text { with } n \in \{1,\ldots ,N\}, \, g \in G \end{array}\right. } \end{aligned}$$

11 $$\begin{aligned} \rho (x,t)&= {\left\{ \begin{array}{ll} \rho _0, &{} x \in {\mathbb {R}}^2 \setminus \overline{{\mathcal {D}}}, \\ \rho _n(t), &{} x\in g + D_n,\text { with } n \in \{1,\ldots ,N\}, \, g \in G. \end{array}\right. } \end{aligned}$$

That is, the density $\rho$ and the bulk modulus $\kappa$ are piecewise constant in space and also in time in the domain of the background material. However, the material parameters are time-dependent inside the resonators. The goal is to study subwavelength solutions to the wave equation with time-dependent coefficients

12 $$\begin{aligned} \left( \frac{\partial }{\partial t } \frac{1}{\kappa (x,t)} \frac{\partial }{\partial t} - \nabla _x \cdot \frac{1}{\rho (x,t)} \nabla _x\right) u(x,t) = 0, \quad x\in {\mathbb {R}}^2,\, t\in {\mathbb {R}}. \end{aligned}$$

To this end we will restrict ourselves to the study of its associated *subwavelength quasifrequencies*, when $\kappa$ and $\rho$ are *T*-periodic in time, in which case we understand the following by subwavelength quasifrequencies.

When the wave equation (12) is periodic in time and space, one can apply the Floquet transform with respect to time and space to it. This leads to a parameterized set of problems with restricted solution spaces. Indeed, one obtains

13 $$\begin{aligned} {\left\{ \begin{array}{ll}\ \displaystyle \left( \frac{\partial }{\partial t } \frac{1}{\kappa (x,t)} \frac{\partial }{\partial t} - \nabla _x \cdot \frac{1}{\rho (x,t)} \nabla _x\right) u(x,t) = 0,\\ \ u(x,t)e^{-i \omega t} \,\,\hbox { is}\,\, T \hbox {-periodic in}\,\, t, \\ \ u(x,t)e^{-i \alpha \cdot x} \hbox { is}\,\, G \hbox {-periodic in}\,\, x, \end{array}\right. } \end{aligned}$$

where $\omega$ ranges over the elements of the *time-Brillouin zone* $Y_t^*:= {\mathbb {C}} / (\Omega {\mathbb {Z}})$ with $\Omega$ being the frequency of the material parameters, which is thus given by $\Omega = {2\pi }/{T}$, and $\alpha$ ranges over elements of the *Brillouin zone* ${\mathbb {R}}^2/L$. If a solution *u*(*x*, *t*) to (13) exists for an $\omega \in Y_t^*$ and $\alpha \in {\mathbb {R}}^2/L$, then *u*(*x*, *t*) is called a *Bloch solution* and $\omega$ its associated *(time-)quasifrequency* and $\alpha$ its associated *(spatial) quasifrequency*.

In order to study *subwavelength* (time-)quasifrequencies, one needs to assume that the *contrast parameter*

$$\begin{aligned} \delta := \frac{\rho _i(0)}{\rho _0} \end{aligned}$$

is small,^2^ or, to be precise, one needs to consider solutions to the wave equation (12) as $\delta \rightarrow 0$. Assuming

14 $$\begin{aligned} \rho (x,t) = {\left\{ \begin{array}{ll} \rho _0, &{} x \in {\mathbb {R}}^2 \setminus \overline{{\mathcal {D}}}, \\ \rho _r\rho _n(t), &{} x\in g + D_n,\text { with } n \in \{1,\ldots ,N\}, \, g \in G, \end{array}\right. } \end{aligned}$$

such that with $\rho _n(0) = 1$ for all $n = 1,\ldots , N$ and $\rho _r > 0$, one can regard the wave equation (12) as parameterized by the contrast parameter $\delta$ and one can consider its solutions as $\delta \rightarrow 0$. This is called the *high contrast regime*. In the setting where the modulation frequency $\Omega$ may also depend on $\delta$, subwavelength frequencies are introduced as in [5] and are given by the following definition.

#### Definition 6

(*Subwavelength quasifrequency*) A quasifrequency $\omega = \omega (\delta ) \in Y^*_t$ of (13) is said to be a *subwavelength quasifrequency* if there is a corresponding Bloch solution *u*(*x*, *t*), depending continuously on $\delta$, which can be written as

$$\begin{aligned} u(x,t)= e^{i \omega t}\sum _{m = -\infty }^\infty v_m(x)e^{i m\Omega t}, \end{aligned}$$

where

$$\begin{aligned} \omega (\delta ) \rightarrow 0 \in Y_t^* \text { and } M(\delta )\Omega (\delta ) \rightarrow 0 \in {\mathbb {R}} \text { as } \delta \rightarrow 0, \end{aligned}$$

for some integer-valued function $M=M(\delta )$ such that, as $\delta \rightarrow 0$, we have

$$\begin{aligned} \sum _{m = -\infty }^\infty \Vert v_m\Vert _{L^2(D)} = \sum _{m = -M}^M \Vert v_m\Vert _{L^2(D)} + o(1). \end{aligned}$$

In [5], a capacitance matrix formulation to the subwavelength quasifrequencies as $\delta \rightarrow 0$ was proven. It describes subwavelength quasifrequencies as solutions to a (finite dimensional) linear system of equations in the setting where the density and bulk modulus are constant with respect to time. In the setting of time-dependent density and bulk modulus, the subwavelength quasifrequencies are described by a periodically parameterized lODE.

In order to state this result, we need the following definitions of the *time-dependent contrast parameters*, *wave speed* and *time-dependent wave speeds*

$$\begin{aligned} \delta _n(t) = \frac{\rho _n(t)}{\rho _0}, \quad v_0 = \sqrt{\frac{\kappa _0}{\rho _0}}, \quad v_n(t) = \sqrt{\frac{\kappa _n(t)}{\rho _n(t)}}, \end{aligned}$$

respectively, with $$n =1,\ldots ,N$$.

#### Theorem 2

Being in the high contrast regime of (12), assume that the material parameter $\kappa$ is given by (10), that $\rho$ is given by (14) and that they satisfy

$$\begin{aligned} \frac{1}{\rho _n(t)} = \sum _{m = -M}^M r_{n,m} e^{i m \Omega t}, \qquad \frac{1}{\kappa _n(t)} = \sum _{m = -M}^M k_{n,m} e^{i m \Omega t}, \end{aligned}$$

for some $M(\delta )\in {\mathbb {N}}$ satisfying $M(\delta ) = O\left( \delta ^{-\gamma /2}\right)$ as $\delta \rightarrow 0$ for some fixed $0<\gamma <1$. Furthermore, suppose that the associated time-dependent contrast parameters, wave speed and time-dependent wave speeds satisfy for all $t\in {\mathbb {R}}$ and $n=1,\ldots ,N$,

$$\begin{aligned} \delta _n(t) = O(\delta ), \quad v = O(1), \quad v_n(t) = O(1) \quad \text {as } \quad \quad \delta \rightarrow 0, \end{aligned}$$

respectively. Then, as $\delta \rightarrow 0$, the subwavelength quasifrequencies $\omega (\delta ) \in Y^*_t$ to the wave equation (12) in the high contrast regime are, to leading order in $\delta$, given by the quasifrequencies of the system of ordinary differential equations in $y(t) = (y_n(t))_n$,

15 $$\begin{aligned} \sum _{m=1}^N C^\alpha _{nm} y_m(t) = -\frac{|D_n|}{\delta _n(t)}\frac{\, \textrm{d}}{\, \textrm{d}t} \left( \frac{1}{\delta _nv_n^2}\frac{\, \textrm{d}(\delta _ny_n)}{\, \textrm{d}t}\right) , \end{aligned}$$

for $n=1,\ldots ,N$, where $C^\alpha =(C^\alpha _{nm})_{n,m}$ denotes the capacitance matrix associated to the infinite periodic structure of the considered metamaterial and the spatial quasifrequency $\alpha \in {\mathbb {R}}^2/L$.

The *capacitance matrix* is a way to encode the geometry of an infinite periodic structure into a square matrix. It has the same dimension as the total number of resonators in a fundamental domain. The capacitance matrix theory in the high contrast regime was developed in [2]. It was first derived using *Gohberg–Sigal theory* and *layer potential techniques* for the finite structure case, where the resonators $D_1,\ldots , D_N$ are not repeated periodically in space, but where the resonator domain ${\mathcal {D}}\subset {\mathbb {R}}^3$ is given by ${\mathcal {D}} = D_1\cup \cdots \cup D_N$. Then, *Floquet-Bloch theory* allowed to extend the results to infinite structures.

One can rewrite (15) into the following system of Hill equations:

16 $$\begin{aligned} \Psi ''(t)+ M(t)\Psi (t)=0, \end{aligned}$$

where the vector $\Psi$ and the matrix *M* are defined as

$$\begin{aligned} \Psi (t) = \left( \frac{\sqrt{\delta _n(t)}}{v_n(t)}y_n(t)\right) _{n=1}^N, \quad M(t) = W_1(t)C^\alpha W_2(t) + W_3(t) \end{aligned}$$

with $W_1, W_2$ and $W_3$ being diagonal matrices with corresponding diagonal entries

$$\begin{aligned} \left( W_1\right) _{nn} = \frac{v_n\delta _n^{3/2}}{|D_n|}, \qquad \left( W_2\right) _{nn} =\frac{v_n}{\sqrt{\delta _n}}, \qquad \left( W_3\right) _{nn} = \frac{\sqrt{\delta _n}v_n}{2}\frac{\, \textrm{d}}{\, \textrm{d}t}\frac{1}{(\delta _nv_n^2)^{3/2}}\frac{\, \textrm{d}(\delta _nv_n^2)}{\, \textrm{d}t}, \end{aligned}$$

for $$n=1,\ldots ,N$$.

In the following we will analyse the introduced topological invariants in the setting of Eq. (16). As a first step however, we will clarify how we compute the Floque normal form in practice.

### Computation of continuous parametrization of Floquet exponents and Floquet modes

Since from a computational point of view, it is costly to compute matrix exponentials, one needs to develop another procedure to work around this problem. Since in the case of the Type I topological invariants, one needs to compute the Floquet exponents and Floquet modes, it is convenient to reuse those for the computation of $\exp (tF_\alpha )$ and, more importantly, for the computation of the Lyapunov transform $P(\alpha ,t):= X_\alpha (t)\exp (-tF_\alpha )$. The details are given in the following lemma.

#### Lemma 4

Let

$$\begin{aligned} \left\{ \frac{d}{dt}x = A(\alpha ,t)x \right\} _{\alpha \in {\mathbb {T}}^d} \end{aligned}$$

be a periodically parameterized periodic lODE and let *T* be the period of $A(\gamma ,\cdot )$. If the monodromy matrix $X_\alpha (T)$ is locally $C^1$-diagonalizable. Let ${\tilde{L}}\subset L$ be a maximal lattice as in Lemma 2 and let

$$\begin{aligned} \Lambda : \; {\mathbb {R}}^d/{\tilde{L}} \longrightarrow {\mathbb {C}}^N \end{aligned}$$

be a continuously differentiable map that associates to each $\gamma \in {\mathbb {R}}^d$ the characteristic multipliers $\Lambda (\gamma )=(\lambda _1(\gamma ), \ldots , \lambda _N(\gamma ))$. Furthermore, denote by

$$\begin{aligned} \eta : \; {\mathbb {R}}^d/{\tilde{L}} \longrightarrow ({{\mathbb {C}}}{{\mathbb {P}}}^{N-1})^N \end{aligned}$$

a continuously differentiable map that associates to each $\gamma \in {\mathbb {R}}^d/{\tilde{L}}$ the eigenspaces $\eta (\gamma )=(\eta _1(\gamma ), \ldots , \eta _N(\gamma ))$, such that $\eta _n(\gamma )$ is the eigenspace corresponding to the eigenvalue $\lambda _n(\gamma )$. Then the (continuously differentiable and periodic) Floquet-Lyapunov decomposition of $X_\gamma (t)$

$$\begin{aligned} X_\gamma (t) = P(\gamma ,t)\exp \left( tF_\gamma \right) \end{aligned}$$

can be computed using the Floquet exponent matrix which satisfies

17 $$\begin{aligned} F_\gamma = V(\gamma ){{\,\mathrm{{diag}}\,}}\left( \frac{1}{T}\Lambda (\gamma )\right) V(\gamma )^{-1}, \end{aligned}$$

with

$$\begin{aligned} V(\gamma ) = \begin{pmatrix} |&{} &{}|\\ v_1(\gamma ) &{}\ldots &{}v_N(\gamma )\\ |&{} &{}| \end{pmatrix}, \end{aligned}$$

where $v_n(\gamma )$ is some generator of the vector space $V_n(\gamma ) = \eta _n(\gamma )$ for $1 \le n \le N$. The Lyapunov transformation can then be computed as

18 $$\begin{aligned} P(\gamma ,t) = X_\gamma (t)V(\gamma ){{\,\mathrm{{diag}}\,}}\left( \exp \left( -\frac{1}{T}\Lambda (\gamma )\right) \right) V(\gamma )^{-1}, \end{aligned}$$

where $\exp$ denotes the ‘usual’ complex exponential function applied to each coordinate of $\Lambda (\gamma )$, and not the matrix analogue.

#### Proof

Equation (17) is simply a diagonalization of $F_\gamma$ and Eq. (18) is due to the fact that

$$\begin{aligned} \exp (-tF_\gamma ) = \exp \left( V(\gamma ){{\,\mathrm{{diag}}\,}}\left( \frac{1}{T}\Lambda (\gamma )\right) V(\gamma )^{-1}\right) = V(\gamma ){{\,\mathrm{{diag}}\,}}\left( \exp \left( -\frac{1}{T}\Lambda (\gamma )\right) \right) V(\gamma )^{-1}. \end{aligned}$$

$$\square $$

The above lemma is particularly useful when one wants to compute the Lyapunov transform numerically. In fact, the procedure we chose for this paper is the following. We computed the fundamental solution $X_\alpha (t)$ using an appropriate numerical method, then we estimated the eigenvalues $\xi _1(\alpha ), \ldots , \xi _N(\alpha )$ with respective eigenvectors $v_1(\alpha ), \ldots , v_N(\alpha )$ of $X_\alpha (T)$ and parameterized them in a continuously differentiable manner. Since, with the notation from Sect. 2.2, $\Xi (\gamma ) = (\xi _1(\gamma ), \ldots , \xi _N(\gamma ))$ relates to $\Lambda (\gamma )$ in the following fashion

$$\begin{aligned} \Xi (\gamma ) = \exp \left( \Lambda (\gamma )\right) , \text { for } \gamma \in {\mathbb {R}}^N/{\tilde{L}} \end{aligned}$$

we took the appropriate logarithm branch for each $\xi _n(\gamma )$. Then, given

$$\begin{aligned} \Lambda (\gamma ) = \log (\Xi (\gamma )), \end{aligned}$$

one can compute $F(\alpha )$ as

$$\begin{aligned} F_\alpha = V(\gamma )\frac{1}{T}\Lambda (\gamma ) V(\gamma )^{-1}, \gamma \in {\mathbb {R}}^d/{\tilde{L}} \end{aligned}$$

and the Lyapunov transform as

$$\begin{aligned} P(\alpha ,t) = X_\alpha (t)V(\gamma )\exp \left( -\frac{t}{T}\Lambda (\gamma )\right) V(\gamma )^{-1}. \end{aligned}$$

### Analysis of topological effects in the setting of high-constrast metamaterials

This section is dedicated to the analysis of the case where the lODE $\frac{d}{dt}X = A_\alpha (t)X$ is parameterized by $\alpha \in {\mathbb {S}}^1$, that is, where the parameter space ${\mathbb {T}}^d$ is of dimension $d = 1$.

We will see that a one-dimensional parameter space considerably restricts the topological properties of the Floquet normal form of the lODE

$$\begin{aligned} \left\{ \frac{d}{dt}X = A_\alpha (t) X\right\} _{\alpha \in {\mathbb {S}}^1}. \end{aligned}$$

It will be proven that in that case only the Type I.a Topological Invariant remains of interest and that the Type II.b topological invariant is uniquely determined by the function $\det (P)/|\det (P) |$. In other words, the Type I.b and the homotopy class of $S(\gamma ,t)$ are automatically trivial whenever the parameter space is equal to ${\mathbb {S}}^1$. Both results are due to the homotopical properties of ${{\mathbb {C}}}{{\mathbb {P}}}^{N-1}$ and ${{\,\textrm{SU}\,}}(N)$, respectively.

Considering the Type I.b Homotopy Class first, the following result holds.

#### Lemma 5

Let

$$\begin{aligned} \left\{ \frac{d}{dt}x = A(\alpha ,t)x \right\} _{\alpha \in {\mathbb {T}}^d} \end{aligned}$$

be a periodically parameterized periodic lODE and let *T* be the period of $A(\gamma ,\cdot )$. If the monodromy matrix $X_\alpha (T)$ is locally $C^1$-diagonalizable for all $\alpha \in {\mathbb {S}}^1$, then the associated lODE

$$\begin{aligned} \left\{ \frac{d}{dt}X = A_\alpha (t)X\right\} _{\alpha \in \mathbb {S}^1\cong \mathbb {R}/L} \end{aligned}$$

has trivial Type I.b Homotopy Class.

#### Proof

Let

$$\begin{aligned} \eta = (\eta _1,\ldots ,\eta _N): \quad {\mathbb {R}} \longrightarrow ({{\mathbb {C}}}{{\mathbb {P}}}^{N-1})^N \end{aligned}$$

be as in Definition 3. That is, let $\eta$ be a continuous lifting of the characteristic multipliers $(\lambda _1,\ldots ,\lambda _N)$. Recall that the Type I.b homotopy class associated to $\lambda _n$ was defined as the homotopy classes

$$\begin{aligned} \eta _n \in [{\mathbb {R}}/{\tilde{L}},{{\mathbb {C}}}{{\mathbb {P}}}^{N-1}] \cong [{\mathbb {S}}^1,{{\mathbb {C}}}{{\mathbb {P}}}^{N-1}], \end{aligned}$$

with $1 \le n\le N$. Since complex projective space ${{\mathbb {C}}}{{\mathbb {P}}}^{N-1}$ is simply connected for $N \ge 1$, it follows that $[{\mathbb {S}}^1,{{\mathbb {C}}}{{\mathbb {P}}}^{N-1}]$ consists of precisely one element and thus the Type I.b Homotopy Class of $\left\{ \frac{d}{dt}X = A_\alpha (t)X\right\} _{\alpha \in {\mathbb {S}}^1}$ is always trivial. $\square$

The argument for the Type II.b Homotopy Class is similar. It relies on the fact that ${{\,\textrm{SU}\,}}(N)$ is 1- and 2-connected for all $N \ge 1$.

#### Lemma 6

Let

$$\begin{aligned} \left\{ \frac{dx}{dt} = A(\alpha ,t)x \right\} _{\alpha \in {\mathbb {S}}^1} \end{aligned}$$

be a periodically parameterized periodic lODE and let *T* be the period of $A(\gamma ,\cdot )$. Assume the setting of Definition 5 and using the notation of Lemma 3. Then the Type II.b Homotopy Class of the associated lODE

$$\begin{aligned} \left\{ \frac{dX}{dt} = A_\alpha (t)X\right\} _{\alpha \in \mathbb {S}^1\cong \mathbb {R}/L} \end{aligned}$$

is uniquely determined by the homotopy class $\Delta \circ P \circ \iota \in \pi ^1({\mathbb {S}}^1)$.

#### Proof

Let *P* be as in Lemma 2 and let $S(\gamma ,{\overline{t}}) \in C^0({\mathbb {R}}/{\tilde{L}} \times {\mathbb {R}}/T{\mathbb {Z}}, {{\,\textrm{SU}\,}}(N))$ be obtained from $P(\gamma ,{\overline{t}})$ as indicated in Eq. (9). Recall that the Type II.b Homotopy Class associated to the lODE $\left\{ \frac{d}{dt}X = A_\alpha (t)X\right\} _{\alpha \in {\mathbb {S}}^1}$ is uniquely determined by the homotopy class $\Delta \circ P \circ \iota \in \pi ^1({\mathbb {S}}^1)$ and the homotopy class of *S* in $C^0({\mathbb {R}}/{\tilde{L}} \times {\mathbb {R}}/T{\mathbb {Z}}, {{\,\textrm{SU}\,}}(N))$. In the case of a one-dimensional parameter space ${\mathbb {S}}^1$, the space $C^0({\mathbb {R}}/{\tilde{L}} \times {\mathbb {R}}/T{\mathbb {Z}}, {{\,\textrm{SU}\,}}(N))$ is homeomorphic to $C^0({\mathbb {T}}^2, {{\,\textrm{SU}\,}}(N))$. However, ${{\,\textrm{SU}\,}}(N)$ is 1- and 2-connected for all $n \ge 1$ and thus every continuous function ${\mathbb {T}}^2 \rightarrow {{\,\textrm{SU}\,}}(N)$ is homotopic to a constant function. In particular, $S \in C^0({\mathbb {R}}/{\tilde{L}} \times {\mathbb {R}}/T{\mathbb {Z}}, {{\,\textrm{SU}\,}}(N))$ is always homotopically trivial. In other words, the Type II.b Homotopy Class associated to the lODE $\left\{ \frac{d}{dt}X = A_\alpha (t)X\right\} _{\alpha \in \mathbb {S}^1\cong \mathbb {R}/L}$ is uniquely determined by $\Delta \circ P \circ \iota \in \pi ^1({\mathbb {S}}^1)$. $\square$

### Type I.a homotopic effects

Applying the above theory to the setting of a hexagonal structure one can observe non-trivial instances of the Type I.a Topological Invariant associated to the corresponding parameterized lODE (16). To the best of our knowledge, non-trivial instances of the Type II.b topological invariant haven’t been observed yet in the setting of high-contrast Floquet metamaterials. This indicates that the Type I.a invariant fully characterises the homotopy class of subwavelength solutions in high-contrast time-dependent metamaterials.

**Fig. 3:**
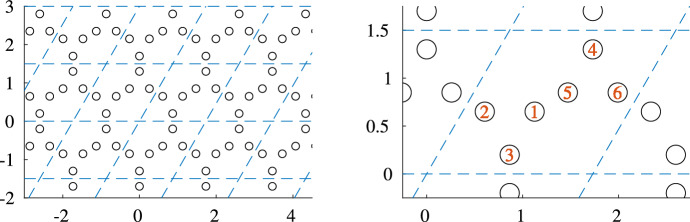
Displayed is the hexagonal structure used in Sect. 3.4. The lattice is displayed with blue dashed lines and the resonators are displayed as black outlined circles. In the right figure, the resonators inside the fundamental domain $\{a_1g_1 + a_2g_2 | a_1, a_2 \in [0,1) \}$ are displayed and numbered according to the notation used in Sect. 3.4. One can distinguish the two trimers: resonators 1, 2, 3 and resonators 4, 5, 6

Two structures of non-trivial homotopy type that are of nontrivial Type I.a will be presented in the following section. The *static* structure will in both cases be given by the same structure depicted in Fig. 3.

It is given by six circular resonators $D_1, \ldots , D_6$ in the fundamental domain $\{a_1g_1 + a_2g_2\, |\, a_1, a_2 \in [0,1)\}$ associated to the lattice vectors

$$\begin{aligned} g_1 = \begin{pmatrix} \sqrt{3} \\ 0 \end{pmatrix}, \quad g_2 = \begin{pmatrix} \sqrt{3}/2 \\ 3/2 \end{pmatrix}. \end{aligned}$$

**Fig. 4:**
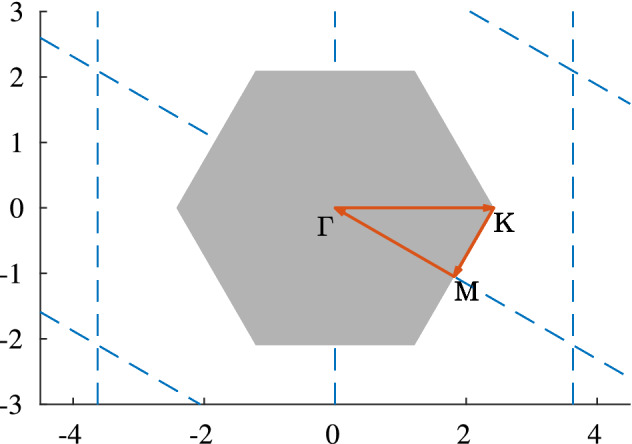
The first Brillouin zone associated to the hexagonal structure used in Sect. 3.4 is colored in grey. On it are displayed the high symmetry points ($~\Gamma$, *K* and *M*) and the (high) symmetry curve, which is indicated with orange arrows. The reciprocal lattice is displayed using blue dashed lines

The resonators have equal radius $R = 0.1$ and are arranged in two trimer blocks (resonators 1, 2, 3 and resonators 4, 5, 6, respectively). The positions of the centers $c_1, \ldots , c_6$ of the six resonators $D_1,\ldots , D_6$ in the fundamental domain are give by

$$\begin{aligned} c_1 = \frac{1}{3}(g_1 + g_2) + 3R\begin{pmatrix} \cos (\frac{\pi }{6}) \\ \sin (\frac{\pi }{6}) \end{pmatrix}, ~ c_2 = \frac{1}{3}(g_1 + g_2) + 3R\begin{pmatrix} \cos (\frac{5\pi }{6}) \\ \sin (\frac{5\pi }{6}) \end{pmatrix}, ~ c_3 = \frac{1}{3}(g_1 + g_2) + 3R\begin{pmatrix} \cos (\frac{9\pi }{6}) \\ \sin (\frac{9\pi }{6}) \end{pmatrix},\\ c_4 = \frac{2}{3}(g_1 + g_2) - 3R\begin{pmatrix} \cos (\frac{9\pi }{6}) \\ \sin (\frac{9\pi }{6}) \end{pmatrix}, ~ c_5 = \frac{2}{3}(g_1 + g_2) - 3R\begin{pmatrix} \cos (\frac{\pi }{6}) \\ \sin (\frac{\pi }{6}) \end{pmatrix}, ~ c_6 = \frac{2}{3}(g_1 + g_2) - 3R\begin{pmatrix} \cos (\frac{5\pi }{6}) \\ \sin (\frac{5\pi }{6}) \end{pmatrix}. \end{aligned}$$

In the case of such a highly symmetric static structure it suffices to consider the capacitance matrix formulation on the *(high) symmetry curve* of the reciprocal lattice *L*. This reduces the a priori 2-dimensionally parameterized system to a 1-dimensionally parameterized system. The new parameterization set is then given by the piecewise linearly interpolated path through the points $\Gamma$, *K* and *M*. It is depicted in Fig. 4.

**Fig. 5:**
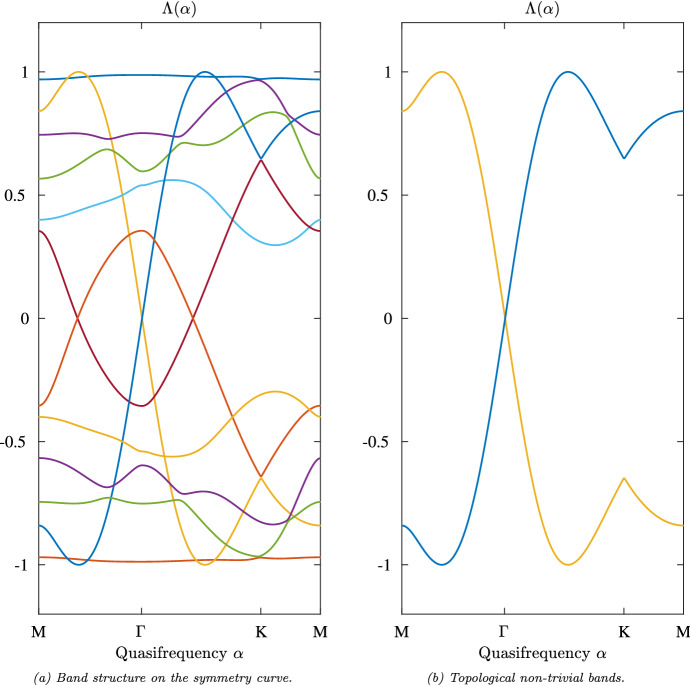
Example of a band structure with non-trivial Type I.a invariant. The band structure of the hexagonal structure presented in this section with modulation given by Eq. (19) and step size equal to $1.21 \times 10^{-2}$, which means 100 steps between *M* and $\Gamma$

By the results of the previous Sect. 3.3, it thus follows that the Type I.b and and the homotopy class of $S(\gamma ,{\overline{t}})$ are always trivial in this case. Hence, the Type I.a Topological Invariant is a good indicator for the different topological nature of subwavelength solutions to the wave equation associated to a time-modulated hexagonal structure. It remains to show that this invariant takes interesting values for some instances of time-modulated hexagonal structure. In the following we will thus present two examples of different instances of the Type I.a Topological Invariant.

The following modulation of the bulk modulus $\kappa$ inside the resonators of the above defined material gives an example of a modulated hexagonal structure where the associated Type I.a Topological Invariant is *non-trivial* and *equal to 2*.

Using the notation from Eq. (10), the following modulation was used

19 $$\begin{aligned} \kappa _1(t) = 1/(1+\varepsilon \cos (\Omega t)),~ \kappa _2(t) = 1/(1+\varepsilon \cos (\Omega t+2\pi /3)),~ \kappa _3(t) = 1/(1+\varepsilon \cos (\Omega t+4\pi /3)),~\end{aligned}$$

20 $$\begin{aligned} \kappa _4(t) = 1/(1+\varepsilon \cos (\Omega t)),~ \kappa _5(t) = 1/(1+\varepsilon \cos (\Omega t+2\pi /3)),~ \kappa _6(t) = 1/(1+\varepsilon \cos (\Omega t+4\pi /3)),~ \end{aligned}$$

where $\Omega = 0.2$ and $\varepsilon = 0.001$. The density $\rho$ was not modulated and set to $\rho _0 = 1$ for the background material and equal to 1/9000 inside the resonators, giving a contrast parameter of $\delta = 1/9000$, ensuring the high-contrast regime.

**Fig. 6:**
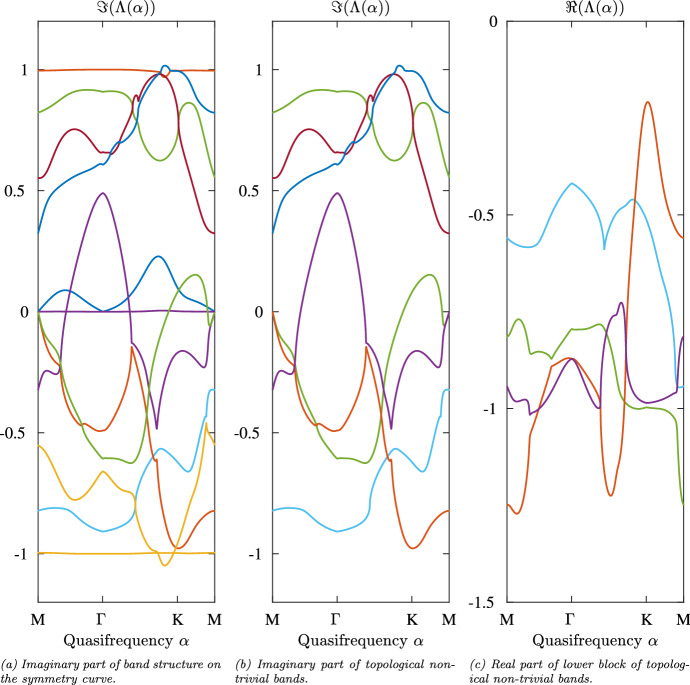
Example of the band structure of a strong modulation with non-trivial Type I.a invariant. The band structure of the time-modulated hexagonal structure with modulation given by Eqs. (21)–(24). The step size was set to $6.05 \times 10^{-3}$, which corresponds to 200 steps between *M* and $\Gamma$. On **a**, the imaginary part of all bands are displayed. In **b** only the imaginary part of those bands which are topologically non-trivial are depicted. This is not evident in the case of the green band. For this reason also the real part of the lower group of topologically non-trivial bands is displayed in **c**

This setting has the associated band structure depicted in Fig. a. Most bands can be glued continuously across the point *M*. However, one band does not connect continuously (see Fig. 5b), but connects with another band which then connects to the former. Thus, the Type I.a Topological Invariant is given by 2 and the structure is topologically non-trivial.

Next, we will present another example of a time-modulation of the hexagonal structure displayed in Fig. 3. This example has the same periodicity in space as well as in time (i.e., the same frequency) as the first example. However, it has three main differences. First, not only the bulk modulus $\kappa$ but also the density $\rho$ is modulated within the resonators, second, a considerably stronger modulation is used. When in the former example a modulation amplitude of $\varepsilon = 0.001$ was used, in the following example the modulation will be 300–500 times larger, having an amplitude of $\varepsilon _\kappa = 0.5$ for the bulk modulus $\kappa$ and an amplitude of $\varepsilon _\rho = 0.3$ for the density $\rho$. Third, the phase shifts of the modulations of the resonators are different.

Using the notation from Eq. (10), the following modulation of the bulk modulus $\kappa$ was used:

21 $$\begin{aligned} \kappa _1(t) = \frac{1}{1+\varepsilon _\kappa \cos (\Omega t)},\quad \kappa _2(t) = \frac{1}{1+\varepsilon _\kappa \cos \left( \Omega t+\frac{2\pi }{3}\right) },\quad \kappa _3(t) = \frac{1}{1+\varepsilon _\kappa \cos \left( \Omega t+\frac{4\pi }{3}\right) },\end{aligned}$$

22 $$\begin{aligned} \kappa _4(t) = \frac{1}{1+\varepsilon _\kappa \cos \left( \Omega t+\frac{2\pi }{3}\right) },\quad \kappa _5(t) = \frac{1}{1+\varepsilon _\kappa \cos (\Omega t)},\quad \kappa _6(t) = \frac{1}{1+\varepsilon _\kappa \cos \left( \Omega t+\frac{4\pi }{3}\right) }, \end{aligned}$$

where $\Omega = 0.2$ and $\varepsilon _\kappa = 0.5$. The shape of the modulation is different from the first example, for this example, it is *mirror-symmetric* between the two trimers.

Defining the modulation of the density, using the notation from Eq. (10), the following modulation of the density $\rho$ was used

23 $$\begin{aligned} \rho _1(t) = \frac{1}{1-\varepsilon _\rho \cos (\Omega t)},\quad \rho _2(t) = \frac{1}{1-\varepsilon _\rho \cos (\Omega t+2\pi /3)},\quad \rho _3(t) = \frac{1}{1-\varepsilon _\rho \cos (\Omega t+4\pi /3)}, \end{aligned}$$

24 $$\begin{aligned} \rho _4(t) = \frac{1}{1-\varepsilon _\rho \cos (\Omega t+2\pi /3)},\quad \rho _5(t) = \frac{1}{1-\varepsilon _\rho \cos (\Omega t)},\quad \rho _6(t) = \frac{1}{1-\varepsilon _\rho \cos (\Omega t+4\pi /3)}, \end{aligned}$$

where $\varepsilon _\rho = 0.3$. Note, that also $\rho$ was chosen to be mirror-symmetric between the two trimers.

This choice of modulation leads to the band structure depicted in Fig. a. Its instance of the Type I.a Topological Invariant is non-trivial, in fact, it is given by 12. In Fig. 6b the two groups of non-trivial bands are displayed. The first group (see the top part of Fig. 6b) being of multiplicity 3 and the second group (see the lower part of Fig. 6b, c) is of multiplicity 4, leading to a Type I.a Topological Invariant of 12. In order to identify the multiplicity of the second group it is necessary to consider the real part of the bands, which are displayed in Fig. 6c. There, it becomes apparent that the purple band is continued by the green band which in turn is continued by the orange band.

## Concluding remarks

In this paper, a topological characterization of the time-local and time-global behaviors of the fundamental solutions of periodically parameterized periodic lODEs was introduced (see Sect. 2). The main starting point of the theory was given by a generalisation of the Floquet normal form of fundamental solutions of periodic lODEs. In fact, a Floquet normal form was introduced, which depends continuously and periodically on the parameter of the considered periodic lODE, allowing for the consideration of homotopy classes of the respective components of the Floquet normal form. Having introduced the respective topological invariants: Type I.a Topological Invariant and Type I.b Homotopy Class for the time-global behavior and Type II.a and Type II.b Homotopy Class for the time-local behavior of the associated parameterized fundamental solution, we were able to perform some analysis on the complexity of the system needed in order to obtain certain topological effects, see Sect. 3.3. Most notably, non-trivial Type I.b Homotopy Classes can only be obtained in the case where the parameter space ${\mathbb {T}}^d$ is of dimension $d \ge 2$. The Type I.a Topological Invariant and the homotopy class of $\Delta \circ P \circ \iota \in \pi ^1({\mathbb {S}}^1)$ being meaningful in the greatest amount of settings, one can use them to topologically distinguish systems with parameter spaces of any dimension $d\ge 1$ and the former is even applicable in the setting of lODEs with constant coefficients.

The implications of this new topological theory for periodically time-modulated hexagonal structures in the high contrast, subwavelength regime are the following (see Sect. 3). Due to the symmetry of a hexagonal structure, some topological effects of the associated subwavelength solutions to the time-modulated material can be distinguished by the Type I.a topological invariant.

We were able to provide two interesting modulation examples of a hexagonal structure which were of non-trivial Type I.a, proving that topologically non-trivial modulations of hexagonal structures do exist. We even provided an example of a relatively high instance of the Type I.a Topological Invariant, the example provided was brading 12 times.

To come back to the original motivation of this paper, namely the analogue of the bulk boundary correspondence in the setting of Floquet metamaterials, a next step would be to built upon the results of this paper and analyse the provided examples for the occurrence of edge modes.

Furthermore, we would like to point out that the developed theory can be refined by considering real-valued coefficient matrices $A: {\mathbb {R}}^d \times {\mathbb {R}} \rightarrow {{\,\mathrm{{Mat}}\,}}_{N \times N}({\mathbb {R}})$ instead of complex valued coefficient matrices. This would particularly enrich the Type II.b topological effects. Indeed, it would mean that the Homotopy Class of $S(\gamma , {\overline{t}})$ would no longer be taken in the space $C^0({\mathbb {T}}^{d+1}, {{\,\textrm{SU}\,}}(N))$ but in the space $C^0({\mathbb {T}}^{d+1}, {{\,\textrm{SO}\,}}(N))$, see e.g. Remark 2. This might provide more topological effects, since ${{\,\textrm{SO}\,}}(N)$ has richer topological properties than ${{\,\textrm{SU}\,}}(N)$, in particular ${{\,\textrm{SO}\,}}(N)$ is not simply connected. This would in particular imply that the *real* Type II.b Homotopy Class also depends on the Homotopy Class of $S(\gamma , {\overline{t}})$, even in the case of a one-dimensional parameter space.

## Data Availability

The datasets generated during and/or analysed during the current study are available from the corresponding author on reasonable request.

## Footnotes

1 In order to avoid pathologies in the case of eigenvalues with geometric multiplicity greater than 1, one should further assume that ${{\,\mathrm{{span}}\,}}_{\mathbb {C}}(\eta _1, \ldots , \eta _N) = {\mathbb {C}}^N$, that is, that the eigenspaces $(\eta _1, \ldots , \eta _N)$ span the whole space ${\mathbb {C}}^N$.

2 Supposing that $\rho _n(0) = \rho _{n'}(0)$ for all $n,n' \in \{1,\ldots ,N\}$.
